# Supplementary figures and images for: Sprouts of Moringa oleifera Lam.: Germination, Polyphenol Content and Antioxidant Activity
Source: Molecules. 2022 Dec 10;27(24):8774. doi: 10.3390/molecules27248774 (PMC9785483; doi:10.3390/molecules27248774)

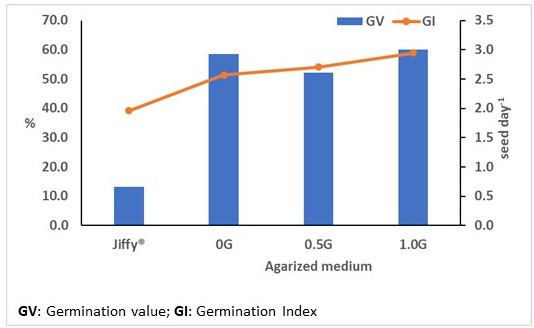

Supplement: Supplementary file 1 [file molecules-27-08774-s001.zip › molecules-2053320-supplementary.jpg]
